# Commodity production as restoration driver in the Brazilian Amazon? Pasture re-agro-forestation with cocoa (*Theobroma cacao*) in southern Pará

**DOI:** 10.1007/s11625-015-0330-8

**Published:** 2015-08-13

**Authors:** Götz Schroth, Edenise Garcia, Bronson Winthrop Griscom, Wenceslau Geraldes Teixeira, Lucyana Pereira Barros

**Affiliations:** 1 C.P. 513, Santarém, Pará 68109-971 Brazil; 2 The Nature Conservancy, Belém, Pará Brazil; 3 The Nature Conservancy, Arlington, VA USA; 4 Embrapa Solos, Rio de Janeiro, Brazil

**Keywords:** Climate-smart commodities, Deforestation, Environmental services, Reforestation, Soil fertility, *Theobroma cacao*, Zero deforestation policy

## Abstract

The increasing demand for agricultural commodities is a major cause of tropical deforestation. However, pressure is increasing for greater sustainability of commodity value chains. This includes the demand to establish new crop plantations and pasture areas on already deforested land so that new forest clearing for agriculture is minimized. Where tree crops are planted as part of agroforestry systems on deforested land, this amounts to a form of re-agro-forestation which can generate environmental benefits in addition to crop production. Here, we discuss a case where agroforestry systems based on cocoa (*Theobroma cacao*) are being established on crop and pasture land in the south of Pará state, Brazilian Amazon. The adoption of cocoa by farmers and ranchers of the region is stimulated by the coincidence of (1) favorable prospects for cocoa on the national and international markets including the expectation of a global cocoa supply gap; (2) environmental policies obliging land owners to reforest excess cleared land with native trees, with agroforests based on the native cocoa tree being an economically attractive option; and (3) biophysical conditions (especially soil fertility) favorable for growing cocoa in part of the region. We show that in the state of Pará at least 1.26 million hectares of naturally high-fertility soils in deforested areas outside legally protected and indigenous lands are potentially suitable for cocoa production with low agrochemical inputs, sufficient to make a significant contribution to closing the predicted supply gap. Their actual suitability depends on their state of degradation after years of pasture use and the availability of technologies and finance to convert them into tree crop agroforests. We discuss the significant environmental benefits of pasture re-agro-forestation with cocoa-based systems, including reduced emissions of up to 135 Mg of carbon per hectare compared to the historically common scenario of planting cocoa after forest clearing. We identify important research questions related to the scaling up of this practice and the maximization of its environmental benefits. We conclude that the coincidence of the afore-mentioned factors could drive a re-agro-forestation frontier in this part of the Amazon, with potential for positive outcomes in terms of commodity production while generating social and environmental benefits.

## Introduction

The ever-increasing demand for agricultural commodities has caused large-scale deforestation in the tropics. Well-known examples include the expansion of oil palm (*Elaeis guineensis*) for cooking oil, biofuel, and a myriad other products that is threatening tropical forests especially in Southeast Asia (Fitzherbert et al. 2008), and the expansion of cattle (*Bos* spp.) pasture and soybean (*Glycine max*) for satisfying the world’s increasing demand for animal products mainly in Latin America, including the Amazon region (McAlpine et al. 2009; Nepstad et al. 2014). Cocoa (*Theobroma cacao*), coffee (*Coffea* sp.) and sugarcane (*Saccharum officinale*) add to the list of tropical commodities that have been implicated in tropical deforestation over the centuries right up to the present (Clay 2004).

Focusing commodity production on already deforested areas is a crucial step in reducing the environmental impact of the current commodity boom and the expected longer-term increase in global demand for tropical agricultural products. Various commodity roundtables, certification systems and corporate sustainability strategies emphasize intensification of commodity production in already deforested areas as part of a strategy to reduce pressure on pristine ecosystems, although these efforts have so far only been partly successful (Millard 2011; Tscharntke et al. 2015). While focusing on deforested land seems straight-forward enough as a strategy for increasing the supply of crops such as oil palm, soybean, coffee, or sugarcane that can easily be planted on old crop or pasture land, for certain tropical commodities it would represent an historic innovation. For example, cocoa has through the centuries mostly been planted on recently cleared or thinned forest land, and aging plantations have usually not been replanted at the same site, but instead been replaced by new farms on newly deforested land (Clarence-Smith 1996). The term “forest rent” has been coined to summarize the various advantages that cocoa farmers have been seeking when planting cocoa on new forest rather than existing farm land (Ruf and Schroth 2004; Ruf et al. 2015). They include fertile soil, protected microclimate and low pressure from weeds, pests and diseases, but also the benefit of not having to cut down old cocoa trees that may still provide a small yield and of laying claim to a new piece of land, often preempting similar claims from others (Ruf and Schroth 2004).

In the case of cocoa, this forest frontier dynamic was mostly driven by small farmers and has not drawn the same public attention as the mostly estate-driven deforestation for some other crops such as oil palm. Nevertheless, cocoa expansion has been a major contributor to millions of hectares of forest loss in regions including West Africa over the past half-century (Gockowski and Sonwa 2011; Ruf et al. 2015). Unsustainable production practices causing low yields and early decline of established farms have aggravated the problem by accelerating the cycle of new planting. Changes in global and national policies that have made it less acceptable to increase commodity outputs at the cost of deforestation, the exhaustion of off-reserve forests in important cocoa producing countries especially in West Africa, and the failure of per-hectare yields to increase sufficiently fast are contributing to fears that in the near future the increase in global cocoa supply may not match the long-term annual increase of 2–3 % in cocoa demand, including from new chocolate consumers in Asia (Lass 2004). Factors conspiring to maintain a flat production curve in important cocoa producing countries include a history of neglect of the smallholder dominated cocoa sector, deficient extension services, aging farms and farmers, high prices for inputs such as fertilizers, and a host of pests and diseases some of which are still in active expansion (Flood and Murphy 2004). The prospect of deteriorating climatic conditions in some of the world’s leading producer countries in West Africa adds to this pessimistic scenario (Läderach et al. 2013). Taken together, these factors have raised the prospect of a cocoa supply gap on the global market of up to 1 million tons over the next decade (Lass 2004; CacaoNet 2012; Dienhart and Mohan 2013). Considering average increases of cocoa demand and per-hectare productivity gains of the last decades, it has been estimated that in order to avoid a supply gap it would be necessary to establish annually 130,000 ha of new cocoa plantations (Mendes and Reis 2013).

At first sight, then, cocoa appears to be just one more commodity for which increasing global demand puts pressure on already embattled tropical forests. However, what cocoa has in common with few other tropical commodities is that it can be grown in forest-like systems (agroforests) where it forms the understorey below a canopy of companion trees (Schroth et al. 2004b; Schroth and da Mota 2014). These trees fulfill a range of functions including shading and microclimatic protection of young cocoa trees, but can also play productive roles (timber, fuelwood, fruits…), maintain soil fertility, store carbon, and host pollinators and predators of cocoa pests (Schroth and Harvey 2007; Tscharntke et al. 2011). Moreover, they provide broader ecosystem services such as increased carbon storage (Schroth et al. 2013), water, energy, and nutrient cycles closer to those of forest ecosystems, and increased biodiversity compared to monoculture systems (Cassano et al. 2009; Tscharntke et al. 2011; Waldron et al. 2012). If cocoa (or any other) agroforests were planted on previously cleared land, this would amount to a form of “re-agro-forestation,” a term coined by Michon et al. (2000) for the re-establishment of forest cover based on useful trees on slash-and-burn land by smallholder farmers in Indonesia. While this has rarely been the case in the history of cocoa cultivation, or commodity production in general, there are reasons to believe that this could now be changing as forest clearing for agricultural expansion becomes increasingly unacceptable (Dinerstein et al. 2014).

Here we look at one area of current cocoa expansion, southeastern Pará state in the Brazilian Amazon. In this vast region now widely dominated by cattle pasture, we may currently be seeing the beginning of a commodity-driven “re-agro-forestation frontier,” brought about by the coincidence of the afore-mentioned prospect of a significant supply gap for cocoa on the global market with a favorable policy and biophysical environment. In this paper, we explore the context, drivers and significance of this process, and discuss potential benefits for global cocoa supply and the environment. We also highlight uncertainties and potential limiting factors for the adoption of cocoa agroforestry among land users in the region, sketch out the research agendas to clarify them and identify policies that could help perpetuate and expand this desirable trend for cocoa and perhaps some other tropical commodities.

## Materials and methods

### Study area and deforestation history

**Fig. 1:**
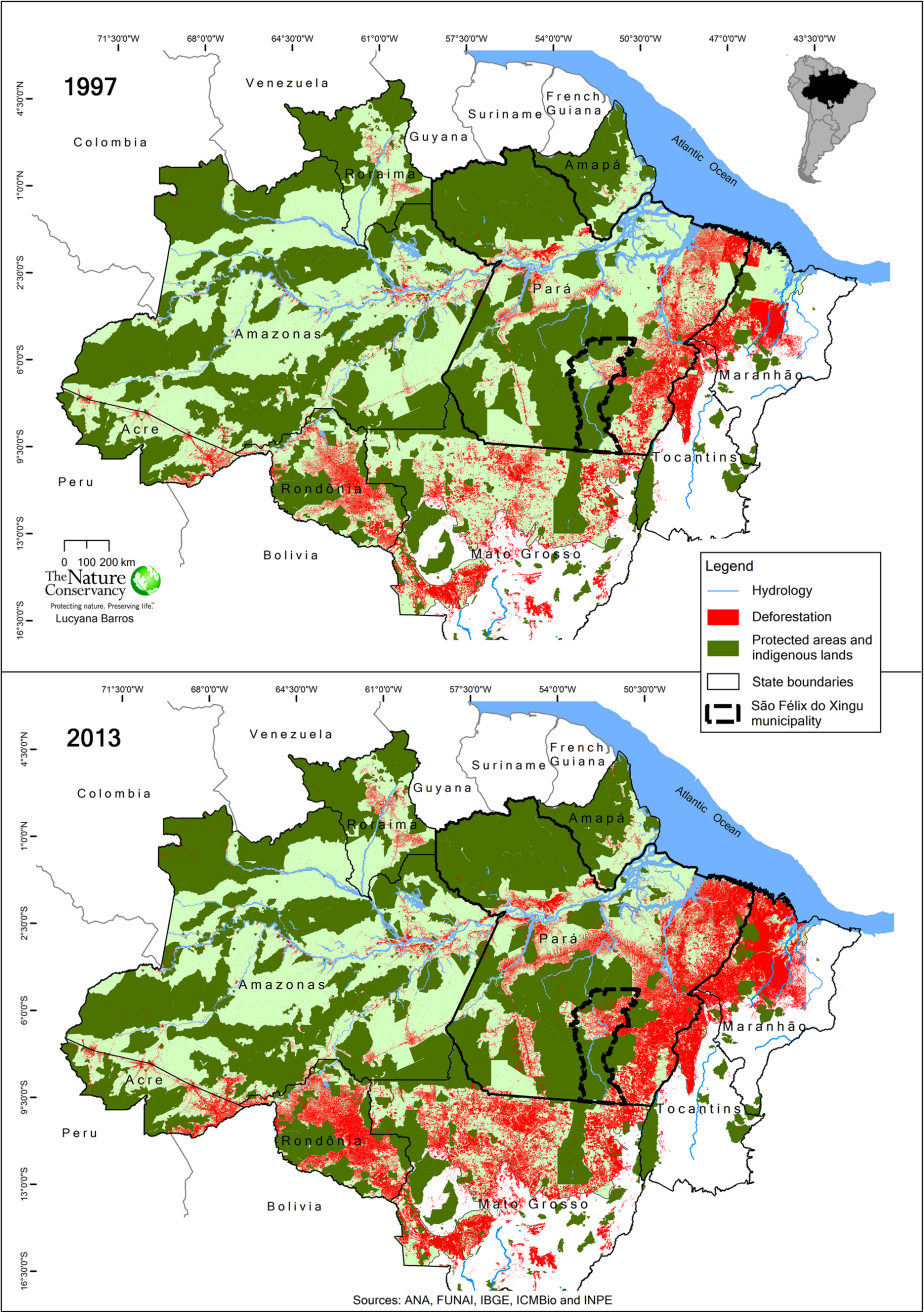
Progressive deforestation in the Amazon biome between 1997 and 2013, including the state of Pará and the municipality of São Félix do Xingu (data downloaded from http://www.obt.inpe.br/prodes/index.php on 24 Jan 2014)

The study focuses on the state of Pará in the northeastern part of the Brazilian Amazon region (Fig. 1). The area has a humid tropical climate, with average annual temperature of 26 °C and annual rainfall generally between 1800 and 2200 mm, conditions favorable to the cultivation of many tropical crops including cocoa (Wood and Lass 2001). It has a relatively pronounced dry season between June and October, and heavier rains occur from December to March. The area is moderately hilly (up to 300 m a.s.l.). The natural vegetation is generally dense upland forest, with areas of open forest in the south (Schneider et al. 2002).

By the year 2014, the Brazilian Amazon had lost approximately 76 million hectares of forest (INPE 2014). Historically, this deforestation has been highly concentrated along the region’s most accessible southern and southeastern edge, a zone known as the “arc of deforestation” that stretches from the states of Acre and Rondônia in the west through the north of Mato Grosso, the south and east of Pará to the western part of Maranhão (Fig. 1). However, the last decades have also seen an increasing expansion of the deforestation frontier into more central parts of the basin, expanding notably along the roads and affecting several protected areas. Deforestation has mostly been driven by the interaction of timber harvesting, slash-and-burn agriculture, and cattle ranching (often taking place in that sequence), as well as more recently the expansion of large-scale, mechanized agriculture, especially for soybean (Fearnside 2001; Laurance et al. 2002; Kirby et al. 2006; Nepstad et al. 2014).

Pará was colonized by Portuguese settlers from the early 17th century (the state capital Belém was founded in 1616), mostly along the Amazon and its tributary rivers, with the native cocoa being its first major agricultural export (Homma 2003). The rubber (*Hevea brasiliensis*) boom of the second half of the 19th and early 20th century gave a major stimulus to the occupation of the region. However, the large-scale occupation of the uplands was initiated by the road construction and planned settlement programs of the Brazilian government starting in the 1960s and 1970s, notably the opening of the Belém-Brasília (north–south) and Transamazon (east–west) highways (Homma 2003; Kirby et al. 2006). The Belém-Brasília highway attracted 2 million settlers during its first 20 years (Kirby et al. 2006). Pará has lost more forest than any other state in the Brazilian Amazon. Deforestation has increased from less than 1 % of Pará’s 1.2 million km^2^ in 1975 to 11 % in 2014 (INPE 1989, 2014), largely due to illegal land speculation and subsequent conversion of forest land for cattle, soybean and subsistence agriculture (Kirby et al. 2006; Rodrigues et al. 2009; Godar et al. 2012).

To illustrate deforestation and land use dynamics that have characterized the forest frontier of Pará and other parts of the Brazilian Amazon, we look in more detail at the example of the municipality of São Félix do Xingu, located within the arc of deforestation in the southeastern part of the state (Fig. 1). With 8,432,811 ha, almost the size of Portugal, it is among the largest municipalities in Brazil. As in other parts of the Amazon, land ownership is highly unequal, with 52 % of the properties having 100 ha or less and covering 6 % of the total area, 38 % of the properties having between 100 and 1000 ha and covering 28 % of the area, and 10 % of the properties having more than 1000 ha and covering 70 % of the area (The Nature Conservancy, unpublished data). More than half (59 %) of the area of the municipality is located within protected areas and indigenous lands, while the unprotected part has seen rapid deforestation and fragmentation due to the expansion of cattle ranching over the past 20 years. When the Brazilian government carried out the first soil survey of the area in the mid-1970s to assess its agricultural suitability, the region was covered by forest that was only used for extractivism of rubber and Brazil nuts (*Bertholletia excelsa*), with a few plots planted with annual crops (SONDOTÉCNICA 1976). Timber (especially mahogany, *Swietenia macrophylla*) extraction from its vast forests followed and cattle ranching soon became the predominant land use on deforested land.

Between 1995 and 2009, the number of cattle in the municipality increased from about 90,000 to over 1.9 million, an over 20-fold increase during a time period when the number of cattle in the whole state of Pará only doubled (IBGE, http://www.ibge.gov.br accessed 07/02/2014). This explosion of cattle numbers reflects the rapid advance of the agricultural frontier as well as the fact that until recently, few other land uses were economically viable in this remote part of the Amazon, encouraging a process called ‘pecuarização” (conversion of other land uses into livestock) in Brazil (Veiga et al. 2004). Field and remote sensing work in the region suggests that cattle pasture continues to be by far the dominant land use in deforested areas, with homegardens and fodder crops (maize) occupying only small areas (The Nature Conservancy, unpublished data).

**Fig. 2:**
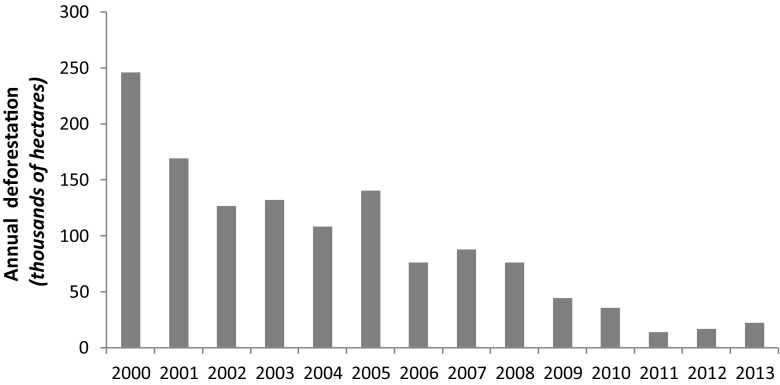
Decrease of the annually deforested area since the year 2000 in the municipality of São Félix do Xingu, southeastern Pará, Brazil, as a combined effect of increased enforcement of environmental legislation and clearing of the most accessible and suitable lands for ranching (data downloaded from http://www.obt.inpe.br/prodes/index.php on 24 Jan 2014)

By 2008, São Félix do Xingu headed a then recently created blacklist of the federal environmental authorities as the municipality with the highest deforestation rate in the entire Brazilian Amazon. This implied that agricultural credit was withheld from land users in this municipality, severely restricting their ability to engage in further farm expansion and land clearing (Nepstad et al. 2014). At that time absolute deforestation rates in the municipality were already in steep decline (Fig. 2). On one hand, this decline suggests that the most accessible and suitable areas had been cleared, although by the year 2013 57 % of private lands outside protected areas in the municipality were still covered by forest fragments (our calculation based on PRODES data). On the other hand, it also indicates that the enforcement of environmental legislation, such as the Brazilian Plan for the Prevention and Control of Amazon Deforestation (PPCDAM), in combination with the remote sensing-based monitoring systems Program for the Estimation of Deforestation in the Brazilian Amazon (PRODES) and Program for Real-Time Detection of Deforestation (DETER) of the Brazilian National Institute of Space Research (INPE), were increasingly taking effect. In addition, between 2009 and 2014, more than 84 % of private properties in São Félix do Xingu became registered in the Rural Environmental Registry (CAR, in Portuguese), one of the mechanisms of environmental regulation that became federal law under the new Forest Code of 2012. This has created accountability for deforestation by linking observed deforestation events to specific properties and their owners, thereby making them liable to prosecution and fines (Nepstad et al. 2014).

### Cocoa in southern Pará

**Fig. 3:**
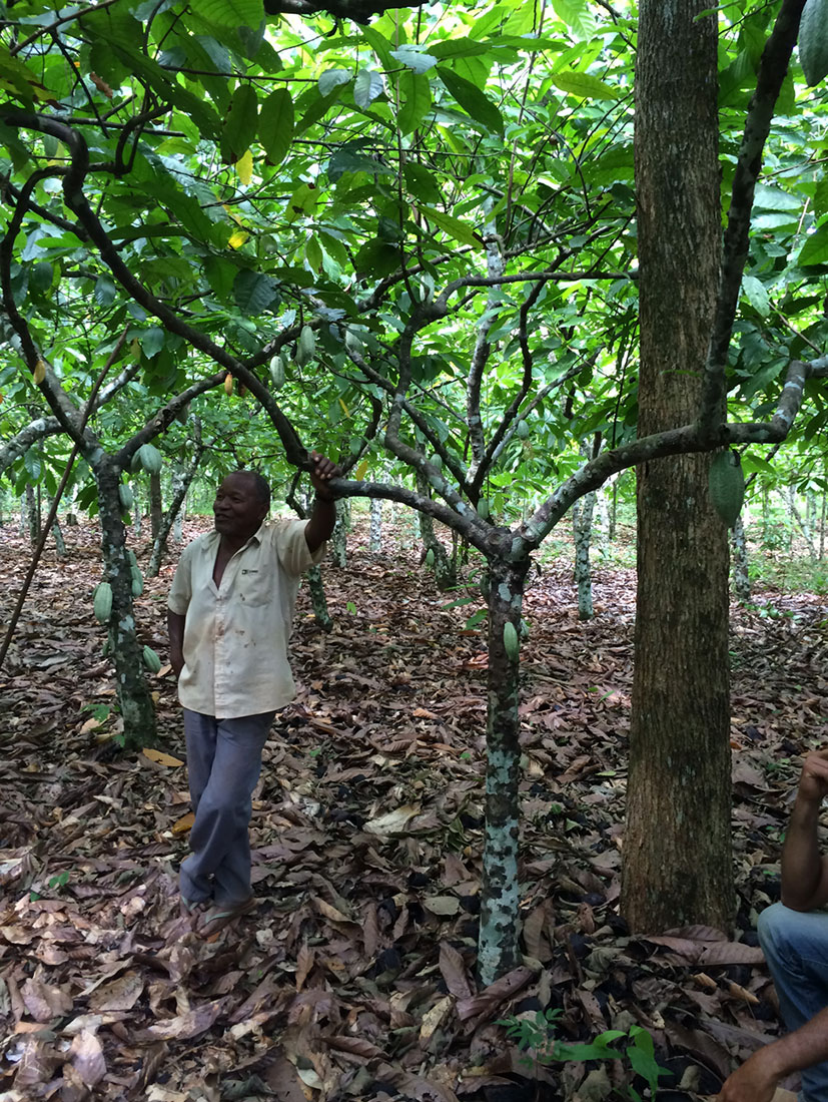
Farmer in his well-managed cocoa agroforest producing an annual harvest of about 2 t ha^−1^ of cocoa in the municipality of São Félix do Xingu, southeastern Pará State, Brazilian Amazon. Although endemic to the Amazon region, there are no signs of the witches’ broom fungus (*Moniliophthora perniciosa*) on the farm, presumably as a result of the still relatively small and fragmented cocoa area interspersed with pasture and the relatively pronounced dry season in this part of the Amazon (Photo: B. Griscom)

While cattle pasture is by far the dominant land use of southern Pará, cocoa has played an important role in the early colonization of the region along the Transamazon highway in the 1970s, where it was grown on basaltic soil locally known as *terra roxa* (Nitossolos) (Veiga et al. 2004). Cocoa has also been promoted through government projects in several parts of Pará as alternative land use and diversification option especially for smaller land owners (Mendes 2014). Currently the area planted to cocoa in the state is over 140,000 ha with a total annual production of 88,000 tons (Martins et al. 2013), and the establishment of another 120,000 ha in the state are planned under a government program by 2022 (Mendes and Reis 2013). While most of the current output is still produced along the Transamazon highway, where cocoa is now expanding on land previously used for sugarcane (Godar et al. 2012), cocoa has also become the second most important agricultural product after cattle in the municipality of São Félix do Xingu further to the south. Here, in 2011 the area planted to cocoa was about 6,000 ha of which almost 60 % were recent plantings not yet in production. The neighboring municipality of Tucumã had an area of 7,455 ha of cocoa planted with 27 % not yet in production. These numbers reflect the rapid expansion of cocoa on soils of naturally high fertility (Mendes 2014). Yield levels of 1–2 t ha^−1^ are reached on these soils with little fertilizer inputs (Fig. 3). Preference given by farmers in the region to those high-fertility soils for cocoa planting is partly reflected in an average cocoa yield of 850 kg ha^−1^ in the whole Amazon region, including areas with less fertile soils (Mendes and Reis 2013), as compared to typical yields of 250–300 kg ha^−1^ in southern Bahia, Brazil’s most important cocoa producing region on the Atlantic coast (Midlej and Santos 2012; Schroth et al. 2014).

### Legal-political factors favoring cocoa planting

While the suitability of parts of the uplands of Pará for cocoa planting had been known since the early days of colonization in the 1970s (Veiga et al. 2004; Mendes 2014), a factor that has recently sparked interest in cocoa planting among land owners, non-government organizations, government agencies, and a few private companies in the region is the coincidence of legal-political factors with the afore-mentioned prospect of increasing cocoa prices due to an increasing supply gap on the world cocoa market. Under the Brazilian Forest Code, 50–80 % of each property in the Amazon—depending on location and time when deforestation occurred—must be kept under native forest cover as “Legal Reserve (LR)” where economic uses under a sustainable management plan are permitted. Until recently, this limit to deforestation was routinely ignored. Under the new Forest Act of 2012, however, pressure to comply has increased (Nepstad et al. 2014), and many land owners now need to bring illegally converted land back under forest cover. In the municipality of São Félix do Xingu, for example, over 70 % of the properties have deforested more than permitted by the Forest Code (The Nature Conservancy, unpublished data) and areas converted after July 2008 are now required to be restored to native tree vegetation, while illegal deforestation that occurred before July 2008 may be offset by others mechanisms. The area to be restored also includes Areas of Permanent Preservation (APP), located along watercourses, around springs and on steep slopes. Whereas the LR can be located flexibly within a farm or even be offset in another place, a degraded APP needs necessarily to be restored in situ. For family farms, requirements are more lenient than for large farms, permitting agroforestry including exotic species in both LR and APP.

**Fig. 4:**
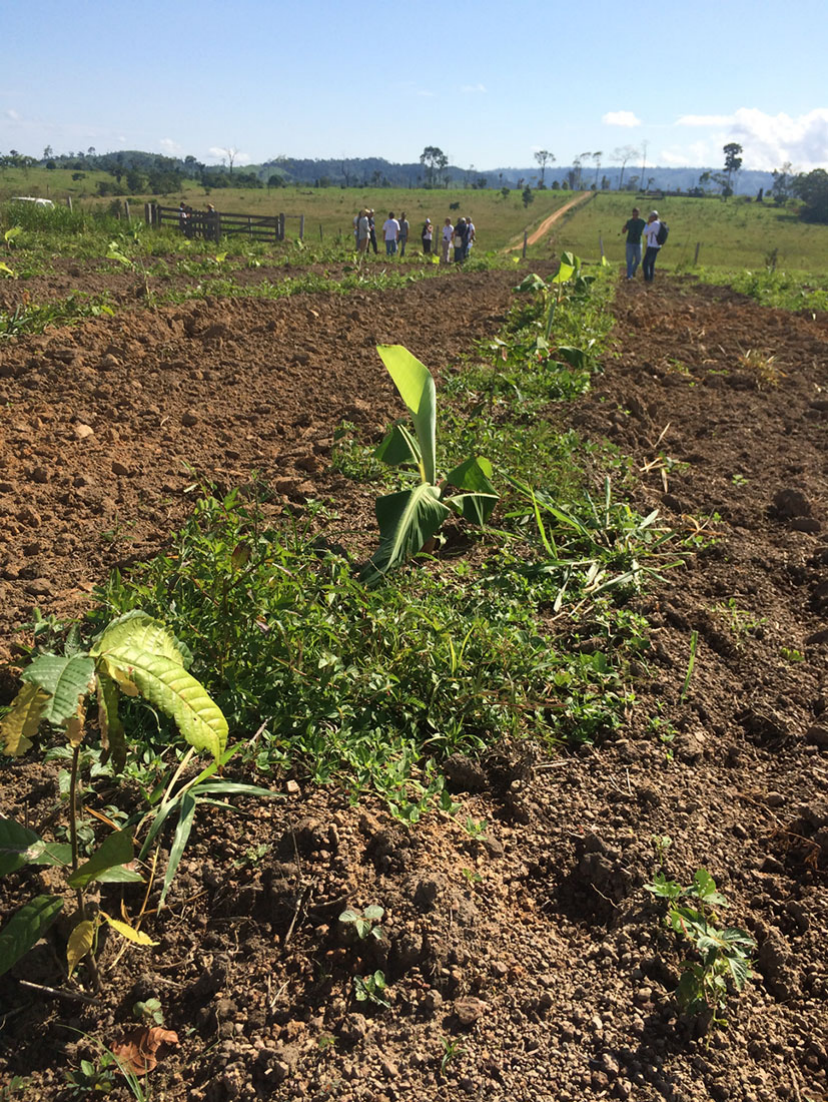
Former pasture land being reforested with a mixture of cocoa (*Theobroma cacao*) trees and bananas (*Musa* sp.), with timber trees to be integrated later, in the municipality of São Félix do Xingu, southeastern Pará State, Brazilian Amazon. The area was plowed to remove vegetation and topsoil compaction (Photo: B. Griscom)

Cocoa being a native tree of the Amazon forest, the law recognizes mixed plantings of cocoa with other native trees (effectively, cocoa agroforests) as permissible vegetation for restoration. Therefore, properties with excess deforestation can now be legalized by “re-agro-foresting” them with a mix of cocoa and other native trees. This prospect has created a strong demand among land owners for cocoa planting material from the government agency in charge of cocoa, CEPLAC (Mendes 2014). It has also attracted the interest of commodity traders (Dienhart and Mohan 2013) and environmental organizations that see opportunities to pursue, respectively, their objectives of increasing the cocoa supply on the national and global markets and the restoration of (agro)forest cover on former pasture or crop land (Fig. 4). The latter group includes The Nature Conservancy, which has been promoting the restoration of degraded pastures with cocoa agroforestry systems in small farms and improvement of pasture management in medium and large properties in the municipality since 2011, in partnership with local and federal organizations as well as the private sector.

### Soil suitability classification

We wanted to know how much land was potentially available in Pará for planting cocoa agroforests without causing deforestation. Cocoa can grow on a wide range of soils, depending on the amount of agrochemical inputs used (Wood and Lass 2001; Neto et al. 2013). Most of the Amazon region is covered with soils of low fertility and this is generally considered a limiting factor in its agricultural development (Schneider et al. 2002). To suit a demanding crop such as cocoa, those soils would require regular additions of mineral fertilizers. Since chemical fertilizers and lime are expensive in the Amazon owing to the transport distances from mines and factories (Mendes and Reis 2013), we were interested in those soils where cocoa can successfully be grown with little or no agrochemical inputs. This is the case for most soils that are characterized as *eutrophic*, which means that at least half of their cation exchange complex (largely made up of organic matter and clay minerals) is occupied by nutrient cations, especially calcium, magnesium, and potassium (“bases”), rather than acidic aluminum and hydrogen ions (“acidity”). We identified the area with such high base status soils on the soil map of Brazil (Santos et al. 2011). This map was created through radar, aerial photographs, and ground truthing in the 1970s through the RADAMBRASIL project. The soil maps were originally published at the scale of 1:1,000,000 and then compiled to the scale of 1:5,000,000. The map distinguishes 244 soil mapping units for the Amazon region. Each unit corresponds to a soil association which is composed of one dominant and up to three secondary soil types, as explained below. We excluded high base status soils if they were shallow and stony and might therefore be seasonally too dry for cocoa [*Neossolos Litolicos* in Brazilian soil classification (Embrapa 2013), corresponding to Regosols in the World Reference Base Classification (WRB 2014)], or if they had anoxic characteristics in the subsoil (*Gleissolos* in the Brazilian soil classification and Gleysols in WRB (2014). The main soil types included as suitable for cocoa owing to their nutrient status and lack of physical restrictions were thus eutrophic subtypes of *Argissolos*, *Neossolos Fluvicos*, and *Nitossolos* in Brazilian soil classification, corresponding mainly to Acrisols, Fluvisols, and Nitisols, respectively, in the international classification (WRB 2014) (Table 1).

**Table 1:** Main soil types with naturally high suitability for producing cocoa (*Theobroma cacao*) at low input level in Pará state, Brazilian Amazon

Soil type (Brazilian classification)	Soil type (World reference base classification)	Main characteristics	Approximate deforested area in Pará^a^
Neossolos Fluvicos Eutroficos	Eutric Fluvisols	Soil formed from fluviatile sediments with a base saturation >50 %	About 51,000 ha, associated with waterlogged soils in the Amazon flood plain
Nitossolos Vermelhos Eutroficos	Eutric Nitisols	Deep, well drained, red soils with >350 g kg^−1^ of clay and a base saturation >50 %	About 323,000 ha
Argissolos Vermelhos and Vermelho-Amarelos Eutroficos	Eutric Acrisols	Soils whose clay content increases with depth with a base saturation >50 %	About 887,000 ha

^a^Including other soil types of lesser importance with high suitability for cocoa in the same mapping unit

In the soil map of Brazil, soil types that occupy less than 20 % of the area of a mapping unit are not listed in the legend among the secondary soil types. We therefore assumed conservatively that each mapping unit, even those dominated by suitable soils, had 10 % of its area occupied by unsuitable soils and generally discounted this percentage of the area. Soil types that occupy at least 20 % of an area but whose distribution is too small-scale to be mapped out as a separate mapping unit are listed as secondary soil types after the dominant type in the legend. Where secondary soil types were listed, we assumed that each of them occupied 20 % of the area of the mapping unit, unknown unsuitable types occupied 10 % of the area as above, and the remainder was occupied by the dominant type. This is a conservative assumption, because eutrophic soil types were more often present as secondary than as dominant soil types and assuming a higher area percentage than the minimum for the secondary types would therefore have resulted in a larger estimate of the total area of fertile soils. We then estimated the total area of suitable soils from the total area of each mapping unit. We subtracted those areas that were inside indigenous lands or protected areas, with the exception of the “Environmental Protection Area” (*Área de Proteção Ambiental*—APA) category where land use is permitted with certain restrictions. Further, we excluded areas that had not previously been deforested and were assumed to be still covered by natural forest.

### Deforestation and land use mapping

The deforested areas up to 2013 were obtained from the Amazon Deforestation Monitoring Project of the Brazilian government (*Projeto de Monitoramento do Desflorestamento na Amazônia Legal*—*PRODES*) (http://www.obt.inpe.br/prodes/index.php, see Fig. 1). It started in the 1980s by distinguishing forest from deforested areas using Landsat satellite images (30 m resolution). While PRODES has no explicit definition of forest and includes a wide range of tree dominated vegetation types in the Amazon as forest, the Brazilian Government defines forest as “land spanning more than 0.5 ha with trees higher than 5 m and a canopy cover of more than 10 %, or trees able to reach these thresholds in situ” (FAO 2009). Deforestation is mapped annually, and newly deforested areas are added to the already cleared area. In the PRODES system, areas once classified as deforested are kept as deforested, even if they were later abandoned and potentially developed secondary forest cover. In order to avoid overestimation of deforested areas and to identify their destination, we used data of the TerraClass 2010 project (http://www.inpe.br/cra/projetos_pesquisas/terraclass2010.php) which maps land use in areas detected by PRODES at 1:100,000 scale. Areas classified as pasture were then intersected with areas with high-fertility soils as defined previously. All geo-processing and spatial analyses were conducted using an advanced license of the ArcGIS 10.2 software for desktop (http://www.esri.com/software/arcgis/arcgis-for-desktop).

The quantification of deforested, high-fertility areas by way of overlaying soil and deforestation maps is subject to a certain error because, in a soil mapping unit that is only partly composed of high-fertility soils and only partly deforested, we have no way of knowing whether the two areas coincide and therefore assume that high-fertility soils occur in the deforested part of the soil mapping unit in the same proportion as they occur in the mapping unit as a whole. This is conservative, because it is very likely—and often obvious—that in a landscape with several soil types differing in fertility the more fertile soils (often concentrated in the valleys) are deforested first for agriculture and are therefore over-represented in the deforested areas, while infertile soils (including sloping and hilltop areas) are more likely to be under-represented in the deforested areas.

### Estimation of carbon benefits

We illustrate the potential climate benefits at national (or Amazon region) level of pasture re-agro-forestation with cocoa by estimating the carbon flux of locating cocoa agroforests on existing pasture lands as compared with the carbon flux of the “business as usual” (BAU) historic pattern of clearing forest in order to plant cocoa (Table 2). In using this counterfactual scenario, our estimate offers an upper level of the mean carbon additionality value per unit area. Depending upon the specific location and context, and the evolution of BAU scenarios, carbon additionality could be substantially less than our estimate. Likewise, we do not suggest that in the absence of cocoa planting on pasture, cocoa would necessarily become a major deforestation driver in Brazil, although with a continuing increase in market demand and price it could (again) become one, especially in other countries were tree crop farming is currently expanding and environmental policies comparable to those in Brazil are not in place or not being enforced. We also emphasize that for some geographies the additionality of avoiding conversion of forests for establishing new cocoa agroforests may be limited by recent success in reducing deforestation, in which case our upper level estimate does not apply.

**Table 2:** Pathways for cocoa re-agro-forestation of pasture land to reduce greenhouse gas emissions. For details on calculations and references see Methods section

Mechanism	Potential carbon additionality	Assumptions	Reversals
1. Avoided deforestation emissions: Due to reduced loss of native forest converted to cocoa as result of forest protection. Conventional cocoa expansion has caused native forest loss in many parts of the tropics	In the order of 75 Mg C ha^−1^ above and below ground carbon stocks. Calculated as: 135 Mg C ha^−1^ carbon emissions from forest conversion minus carbon stocks of cocoa agroforests established at site, estimated at 60 Mg C ha^−1^. Includes 20 % deduction for heterogeneity in deforestation patterns and incomplete conversion	Forest would not be converted or degraded in the absence of cocoa planting	Market leakage due to displacement of cocoa planting to other forest areas if not applied at national, regional or global scale. Unsatisfied cocoa demand is met through intensification of existing farms
2. Re-agro-forestation sequestration: establishment of cocoa agroforests on previously cleared pasture land	In the order of 60 Mg C ha^−1^ above and belowground carbon stocks, highly dependent on practices used	Cocoa agroforest is managed to reach and maintain time-averaged carbon level of 60 Mg C ha^−1^. No emissions from removal of secondary vegetation. Planted area would not otherwise revert to forest. No net carbon flux from soils	Net emissions could occur if cocoa agroforests displace restoration of native secondary forests which may store more carbon. For displacement of cattle see (5)
3. Avoided fire emissions: better fire management on agroforestry farms to avoid damage to cocoa may reduce escaped fires in nearby native forests	Unknown but could be significant. The extent of escaped fires in the Amazon has high annual variability and can result in 10-80 % emissions of forest carbon stocks (Alencar et al. 2006; van der Werf et al. 2009; Pütz et al. 2014)	Re-agro-forested areas are located in proximity to natural forest, ideally along forest boundaries	None
4. Absorption of avoided deforestation leakage: to the extent that cocoa has larger labor demand per hectare than ranching, cocoa agroforestry can absorb local labor leakage from other avoided deforestation strategies	Unknown, depending on the extent of leakage which can range from <10 % to >90 % (Murray et al. 2004)	Displaced workers from cattle, logging or other sectors linked to deforestation are willing to work in cocoa	If cocoa expansion attracts additional labor to the region, a future slump in the cocoa economy could release labor and trigger additional deforestation
5. Changes in per-hectare emissions of greenhouse gases from agricultural and pasture management (carbon footprint)	Highly dependent on local circumstances. Positive impact if cattle numbers are reduced or management of remaining pasture area intensified	Soil fertility after pasture use is not so strongly degraded as to require large amounts of mineral fertilizer	Fertilizer application to cocoa causes greenhouse gas emissions but is small if naturally fertile soils are planted

We assume a mean aboveground carbon stock of Latin American tropical forests at the deforestation frontier of 137 Mg C ha^−1^ (Baccini et al. 2012), with an adjustment of +23.5 % to include belowground carbon stocks (Mokany et al. 2006), resulting in a total of 169 Mg C ha^−1^. We deduct from this 20 % to account for heterogeneity in deforestation patterns and incomplete emissions of above and below-ground forest biomass during conversion (Morton et al. 2011), resulting in deforestation emissions of 135 Mg C ha^−1^. Some of this carbon would then be sequestered again in the cocoa agroforest growing at the cleared site, or would never be emitted if some forest trees were kept standing as shade trees for the cocoa. Carbon stock data for cocoa agroforestry can vary enormously. In the absence of representative data from cocoa agroforests in the study region where these practices are relatively new and still evolving, we use here an estimated value of 60 Mg C ha^−1^ of above- and belowground carbon based on average data from Somarriba et al. (2013) for cocoa agroforestry systems in Central America. The conversion of forest into cocoa agroforestry would then result in net emissions of 135 − 60 = 75 Mg C ha^−1^. If no cocoa were planted and the forest were conserved, then these emissions would be avoided. If instead the same cocoa agroforest were planted on pasture land, then over time 60 Mg C ha^−1^ would be sequestered at that site. If the system was then not periodically clear-felled and replanted but kept at that carbon level through the periodic harvesting of trees and the continuous rejuvenation of the cocoa, then the total, long-term difference between cocoa planting into forest and on pasture would amount to 75 + 60 = 135 Mg C ha^−1^. This assumes that there are no additional emissions from the removal of secondary vegetation in the pasture areas prior to their replanting with cocoa, because these would be equivalent to the recurring emissions from keeping pasture free of such vegetation, and that the planted area would not otherwise revert to forest, which is in line with historically very low rates of reforestation of deforested areas in the tropics, including Brazil (Hansen et al. 2013). We also assume that leakage from displacement of cattle pasture is avoided by a simultaneous process of pasture intensification as is underway in portions of our study area. Finally, we assume no net carbon flux from soils, given the absence of evidence for such fluxes associated with forest-to-pasture transitions (Murty et al. 2002). These assumptions are the basis for an upper-level mean estimate of carbon additionality per hectare in our study region. Each of them needs to be re-considered at a local level in assessing whether some or all of this potential carbon additionality applies to a specific case of pasture re-agro-forestation.

## Results and discussion

### Extent of area with high-fertility soils

**Fig. 5:**
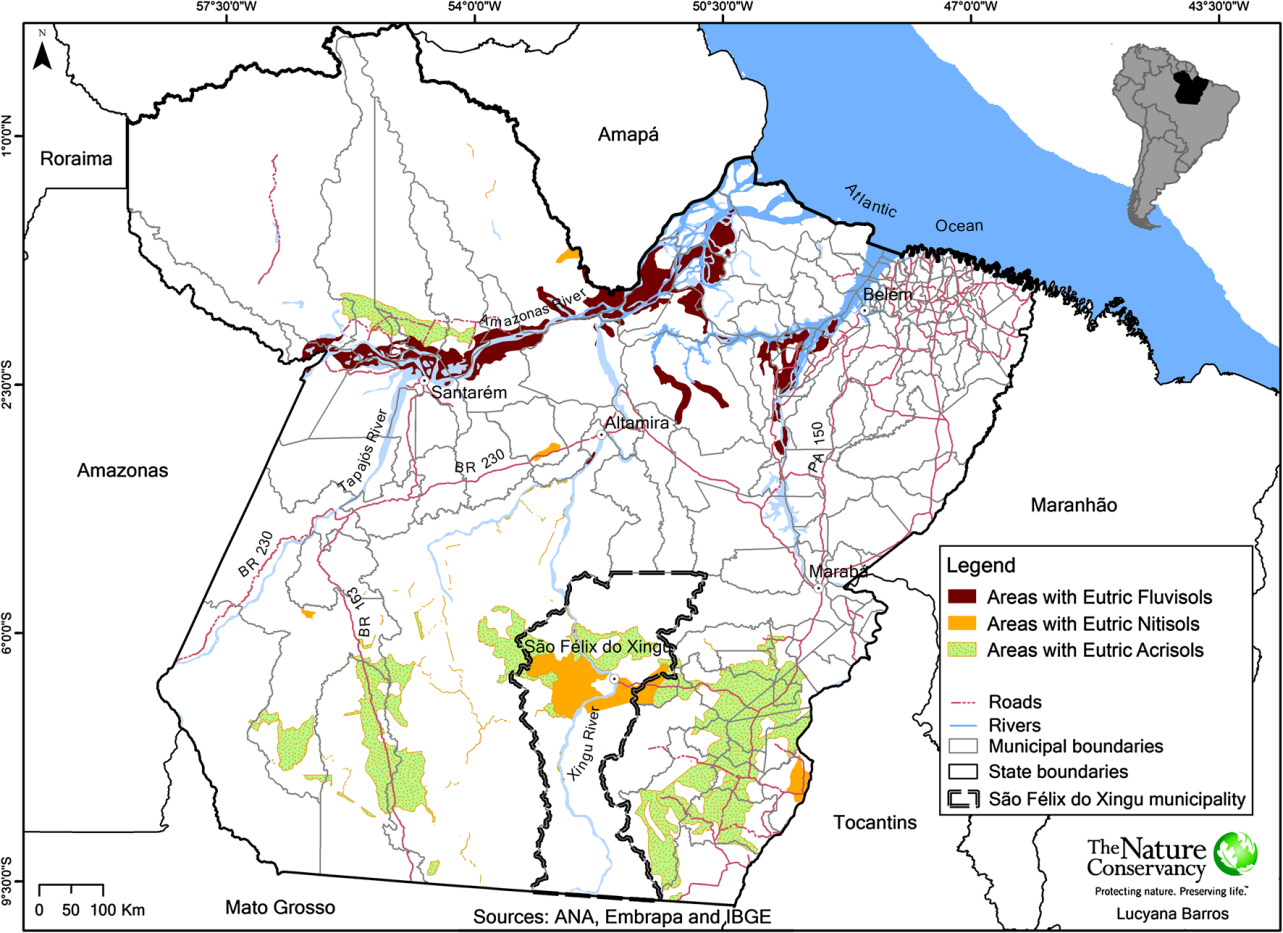
Areas that comprise soils of high chemical fertility potentially suitable for planting cocoa (*Theobroma cacao*) in Pará state, Brazilian Amazon, based on Santos et al. (2011). Only areas outside of indigenous lands and protected areas are shown. Note that high-fertility soils are not always the dominant soil type of the areas shown. Forested and deforested areas are not distinguished on the map, but much of the areas of high-fertility soils are located within the “arc of deforestation” (see Fig. 1)

Our analysis identified deforested areas where naturally fertile soils offer potentially suitable conditions for growing cocoa with low agrochemical inputs in two distinct locations within the state of Pará: in the Amazon floodplain in the northern part of the state, and in the uplands of its southern half, especially the southeast (Fig. 5). Over 600,000 ha of eutrophic alluvial soils, not including waterlogged soils, are located in the Amazon floodplain, but of these only 68,000 ha were classified as deforested (Table 1). The lower Amazon has been an important cocoa producing area in the 18th and 19th century (Alden 1976) but is now dominated by other crops. This area is therefore not further considered here, although its potential to produce again cocoa under current price conditions merits further study.

On the uplands of southern Pará, the area of naturally high-fertility soils was found to be far more expansive. Dominated by eutrophic forms of *Nitossolos* (Nitisols) and *Argissolos* (Acrisols), soils with high chemical fertility, and thus potential suitability for cocoa, were found to cover over 2 million ha in this area, of which 1.26 million ha were classified as deforested (Fig. 5; Table 1). Since cattle pasture is by far the dominant land use in deforested areas in the region, it is safe to assume that most high-fertility soils in deforested areas are also currently under pasture. The potential consequences of prior pasture use for the fertility of these soils are discussed further below.

With current average Amazonian yield levels of 850 kg ha^−1^ (Mendes and Reis 2013), only part of which is produced on high-fertility soils, these 1.26 million ha of deforested land with high-fertility soils would have the potential of producing over 1 million tons of cocoa annually, about a fifth of current global cocoa production (http://icco.org). This total production potential is not likely to be realized any time soon, including because of the negative effect this would have on the world cocoa price. However, these numbers show that over the longer term the expansion potential for cocoa on deforested land in this part of the Amazon is globally significant.

### Adoption of cocoa agroforestry in southern Pará

For a per-hectare yield of 1 t of cocoa currently worth around USD 3000 on the international market, of which a producer in Brazil would receive about USD 2500 (http://icco.org; http://www.ceplac.gov.br), and production costs of USD 750 per ton (Mendes and Reis 2013), estimated annual net profits of fully productive cocoa agroforests are in the range of USD 1750 per ha. This compares to net profits of approximately USD 350 per ha from cattle pasture (The Nature Conservancy, unpublished data), making the adoption of cocoa agroforestry on former cattle pasture economically highly attractive. It is likely that inputs especially of phosphorus fertilizer would be required at the beginning to restore topsoil fertility, depending on the degree of degradation under pasture (Dias-Filho 2011), and this may temporarily reduce profitability. On the other hand, per-hectare yields well above 1 t of cocoa in mature plantations are not unusual on these soils in the region (Fig. 3). Despite the difference in profitability, farmers and ranchers may not put all their suitable pasture land under cocoa because of their traditional connection with cattle which over the last decades has proven the land use with greatest resilience to economic and political crises in the Amazon (Veiga et al. 2004). Furthermore, converting large areas into cocoa would require the hiring of large numbers of workers or sharecroppers (about one per 5 ha of cocoa, as compared to one per several tens of hectares of cattle pasture), who would be costly to train and supervise and not immediately be available in the region. The latter factor may partly explain why in southern Pará cocoa is currently mostly grown on family farms and managed with family labor rather than on large commercial ranches using contract labor. It would, however, be wrong to assume that commercial ranches will not eventually adopt cocoa agroforestry on part of their land if it is seen to be profitable and can help to bring the property into compliance with environmental legislation. Although cocoa is mostly grown by smallholders in Africa, in Latin America including other parts of Brazil, it is also commonly grown on large commercial farms (Schroth et al. 2011). Cultural, economic, and technical obstacles to cocoa adoption in the region and its impacts on the livelihoods of land owners and rural workers require further study.

There may also be biophysical restrictions to cocoa expansion on former pasture land. It is likely that not all soils classified as high-fertility are immediately suitable for cocoa agroforestry after years of pasture use. Pastures in the Amazon are often agronomically degraded through a shift of the vegetation to less palatable species (Dias-Filho 2011), although this would not necessarily affect their suitability for a tree crop like cocoa. In more severe cases of biological pasture degradation reducing the production potential of the area, the soil itself may be negatively affected, including through compaction of the surface horizon and soil erosion on slopes (Dias-Filho 2011). Surface compaction could affect cocoa seedlings through reduced root development and water infiltration (Grimaldi et al. 2003; Germer et al. 2010). Farmers with access to own or rented machinery may solve this problem by plowing the soil (Fig. 4), but smaller farmers may depend on finance to pay for this service. Once covered by tree vegetation, the soil structure would gradually improve through the activities of soil fauna and roots of cocoa and companion trees, much helped by the developing litter layer and avoidance of fire (Cresswell and Kirkegaard 1995; Lavelle et al. 2003; Grimaldi et al. 2003). However, initial tree development could be delayed and in severe cases, increased mortality of seedlings during the dry season could require replanting. After prolonged pasture use without nutrient replacement, the topsoils could also be chemically impoverished, especially in phosphorus, which would have to be corrected through adding mineral fertilizer (Dias-Filho 2011). Soils of naturally high chemical fertility and nutrient stocks are however less susceptible to chemical impoverishment under pasture than naturally infertile soils which are far more common in the Amazon. In the worst cases of soil degradation under pasture, where topsoil fertility has been lost through erosion, the site may not be suitable for a sensitive crop like cocoa unless it is preceded by a fallow phase for soil regeneration. The relative degradation status of naturally high-fertility Amazonian soils under pasture use, the relationships between the degradation status of such soils and their productivity if put under tree crops, as well as cost-effective rehabilitation strategies using or preparing for agroforestry are important areas for future research.

### Environmental benefits of the reforestation of pasture land with cocoa agroforests

Among the environmental benefits generated by the re-agro-forestation of pasture land in the Amazon with cocoa agroforests, we highlight here carbon sequestration or avoided carbon emissions as a global benefit (Table 2). Other environmental benefits that would be felt more locally are briefly discussed at the end of this section. As shown in the Methods section, the national or regional carbon benefit of pasture re-agro-forestation as an alternative to the historical pattern of cocoa planting into newly cleared forest could be as high as 135 Mg C ha^−1^. Locally at the farm scale, these benefits would particularly apply to largely forested farms that still have the option of either legally converting a piece of forest for agriculture or replanting previously cleared pasture land. The avoided deforestation component of these carbon benefits (~75 Mg C ha^−1^) would not apply to many farms in our study region that have already reached or exceeded their legal limits of deforestation. In the following, we discuss various mechanisms through which pasture re-agro-forestation can generate environmental benefits at a generic level, without attempting to quantify these due to the lack of empirical data from the study region and the wide range of possible counterfactual situations against which these would have to be measured.

In climatic as well as biodiversity terms, the most important benefit of cocoa planting on pasture as opposed to the traditional way of planting it into partially or fully cleared forest is the avoided loss or degradation of natural forest. This is because even traditional cabrucas where thinned forest is under-planted with cocoa trees and large forest trees are retained as shade canopy store much less carbon per hectare than does natural forest. For example, the carbon storage in the traditional cabrucas of southern Bahia where most of Brazil’s cocoa is produced is on average only about half that of native forest, and the value is again much lower for intensively managed cabrucas (Schroth et al. 2013). Where cocoa is grown with little or no shade, as in parts of West Africa (Ruf 2011), carbon emissions upon forest conversion into cocoa farms and related biodiversity losses would be even higher.

Instead of causing emissions due to (partial) forest clearing, re-agro-forestation of previously cleared land would result in additional above and belowground carbon storage, especially if high-shade and high-carbon agroforestry practices are used. Carbon stocks in cocoa agroforestry systems vary widely depending on age, composition, and management practices (Somarriba et al. 2013) (“Box 1”). Higher carbon stocks in the vegetation and avoidance of fire can also over time lead to increased soil carbon storage (Gama-Rodrigues et al. 2011), although this outcome is soil specific and difficult to predict or measure (Noponen et al. 2013). These environmental benefits are however dependent on the counterfactual scenario. Not having the option of planting cocoa agroforests, a farmer may allow his excess cleared land to revert to forest which may eventually accumulate higher carbon stocks and be richer in biodiversity. At least in the short term we consider this scenario the least likely for our study region because of the lack of income from such areas, although on the longer term it may become more common as land users are forced to comply with the Forest Code. Even then, a situation where pasture land reverts to secondary forest kept at low biomass level through periodic timber extraction for fence posts, fuelwood or building materials, affected by periodic wild fires, is the most likely. Excess cleared pasture land could also be reforested with plantations of fast-growing native timber trees (Veiga et al. 2004), but this is not currently seen to happen in the region, presumably because of the relatively long financial return period and the cost of bringing large volumes of plantation timber with low unit value to market. Even with the increased pressure to comply with the new Forest Code, we believe that without conservation interventions the most likely scenario would be that land owners would “drag their feet” and delay taking excess cleared land out of pasture use for as long as possible. Efforts to facilitate expansion of high shade cocoa agroforests, by reducing the cost of compliance with the Forest Code, could thus make a critical difference in accelerating the legally required restoration of excess cleared forest land. In line with this notion, some restoration of tree cover has recently been observed in the municipality of Medicilândia, on the Transamazon highway, an area dominated by small to medium sized farms, many of which have fertile *terra roxa* soils and are planting cocoa (Godar et al. 2012).

Farmers opting for cocoa re-agro-forestation will also invest in measures to avoid wild fire damaging their plantation. This mechanism may result in reduced edge effect emissions in adjacent native forests, which can represent 9–25 % of deforestation emissions in fragmented landscapes (Pütz et al. 2014). The role of tree crop agroforestry as an incentive to improve fire control has been highlighted in other regions and with other crops (Schroth et al. 2003, 2009). As a social and environmental benefit, by being relatively labor-intensive, cocoa agroforests may absorb excess labor displaced by the reduction of pasture area, increased control of illegal logging, or other structural adjustments in the local economy, thereby avoiding their displacement to new deforestation frontiers (Angelsen and Kaimowitz 2004).

Finally, if cocoa were planted on less fertile soils that dominate the upland areas in the Amazon region, some savings in greenhouse gas emissions from the afore-mentioned mechanisms would be offset by the emissions from fertilizer application to the cocoa, including the emissions related to transporting fertilizer to remote parts of the Amazon. However, cocoa on the fertile soils considered here requires little fertilizer (Neto et al. 2013), at least once topsoil fertility has been sufficiently restored from its more or less degraded level under pasture to allow cocoa planting. Apart from fertilizer, input-related greenhouse gas emissions from cocoa farming are generally small (Schroth et al. 2014). Cocoa re-agro-forestation could also reduce the number of cattle on the farm and thus methane emissions from the ruminant digestive tract (Gerber et al. 2013). However, since globally the demand for meat and dairy is increasing, this would only mean displacing cattle production to other areas, and so the intensification of the remaining pasture land on the farm or in the landscape to compensate for the area taken out of cattle production is more sensible from a climate point of view (Dias-Filho 2014a). This implies that ideally, farms or municipalities where re-agro-forestation of pasture land with cocoa is taking place should simultaneously invest in pasture intensification, which is a double burden even though specific, presently under-used funding lines of the Brazilian government are available for this (Dias-Filho 2014b).

Significant among non-carbon environmental benefits could be the hydrological services of increased soil and water retention by a forest-like vegetation as compared to open pasture on often sloping soils. Southeastern Pará has a long and pronounced dry season and the hydrological and microclimatic effect of re-agro-forested hill-slopes and riparian buffer strips on the flow and water quality of creeks and springs on which many agricultural and domestic activities depend would surely be felt while these would also act as wildlife corridors (Schroth et al. 2004a). It may be noteworthy that local cocoa varieties of good quality are available that are adapted to flooded soils (Martins et al. 2013) and could be used in the restoration of riparian forests (APP) of which approximately 160,000 hectares need to be restored in São Félix do Xingu (Balieiro et al. 2014). These areas are generally not accounted for by PRODES as deforested because of the small size of individual plots and are therefore mostly additional to the afore-mentioned areas available for cocoa re-agro-forestation. Last but not least, the positive effect of livelihood diversification and increased income for potentially several tens of thousands of landowners, especially those owning relatively small farms for whom livestock can only provide a very modest income (Veiga et al. 2004), also deserves attention.

## Box 1: How to maximize carbon stocks of agroforestry systems while maintaining crop yields

The climate benefits of a cocoa re-agro-forestation strategy on former pasture and crop land depends mostly on technical questions, albeit some non-trivial ones. These include their carbon stocks, the permanence of this storage, and the greenhouse (GHG) emissions from their management relative to pasture. A study in Ecuador found that cocoa yields increased with increasing companion tree density (and presumably carbon stocks) up to an intermediate shade level (Waldron et al. 2012). However, there is little information about the possibility of increasing yields while maintaining system carbon stocks. Recent research in southern Bahia, where more than half of the landscape carbon stocks are contained in traditional cocoa agroforests (Schroth et al. 2013), showed that cocoa yields became depressed if the aboveground carbon stocks in the large companion trees (>30 cm DBH) exceeded 65 Mg ha^−1^, presumably as a combined effect of shade, belowground competition for water and nutrients, and perhaps an increase in disease pressure when stands become too dense and humid (Schroth et al. 2014). Such thresholds need to be established locally since they will depend on soil and climatic conditions as well as yield expectations.

**Fig. 6:**
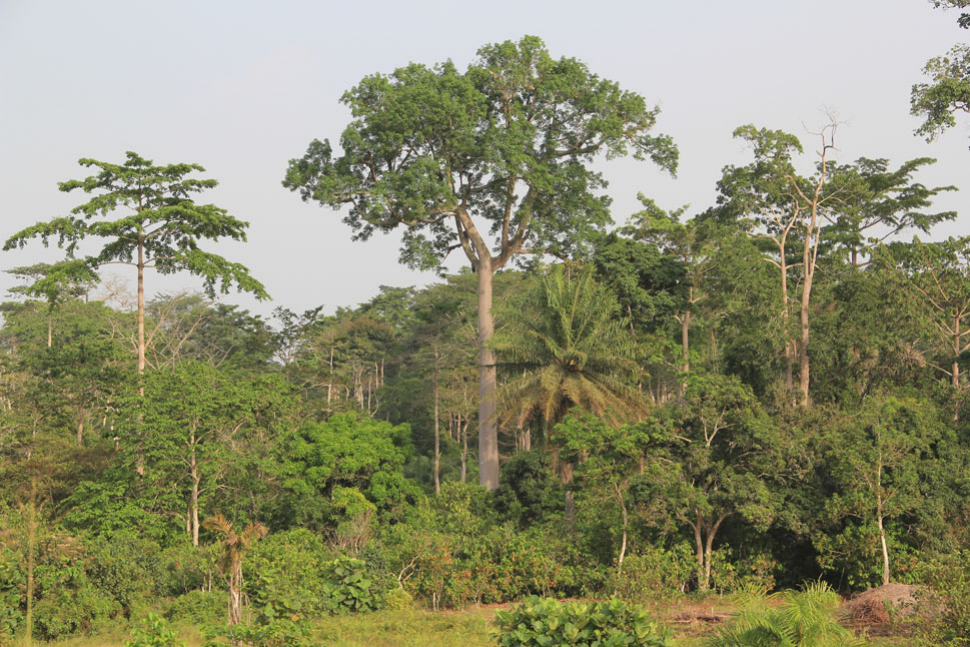
Large trees such as this remnant tree in a cocoa (*Theobroma cacao*) farm in Côte d’Ivoire store more carbon than small trees while interfering less with the light environment of the cocoa trees (photo: G. Schroth)

The shape of the possibility frontier of high carbon stock—yield combinations may also depend on specific tree characteristics. Work in Bahia has established that the carbon stocks of agroforestry systems are highly concentrated in the biggest trees, and it has been suggested that cocoa agroforests could be intensified with little impact on their carbon storage by reducing the density of smaller trees while retaining the big trees whose high crowns interfere less with the light environment of the cocoa trees (Fig. 6) (Schroth et al. 2013).

A further possibility to increase carbon storage in trees without proportionally increasing competition with the crop would be to use trees with particularly high wood density. Reasonably fast-growing species with wood densities around 0.9 g cm^−3^ in this part of the Amazon include *Dipteryx odorata*, a timber and non-timber species, and *Tabebuia* spp., very hardy trees producing export grade timber. The members of another commercially important high wood density group, *Manilkara* spp., are unfortunately very slow growing and therefore less suitable for inclusion in agroforests.

Furthermore, the spatial arrangement of the components could be designed to reduce competition, for example by planting species with dense crowns and competitive root systems, or species that cause excessive damage when harvested, on plot boundaries. Such “box plots” where squares of cocoa are surrounded by rows of leguminous or timber trees have been used successfully in Malaysia (Lim 1980). They are also currently being promoted in Ghana as a means for reducing the spread of the insect vectors of the cocoa swollen-shoot virus, a serious disease of cocoa in West Africa.

Finally, it should be kept in mind that it is not necessarily the highest-producing system that is also the most profitable. A certain level of cocoa yield reduction may be unacceptable to the land owner if it is caused by a tree species with no market value, but not if it is caused by a tree producing fruits or timber for which there is a local market.

## Conclusions

The ever-increasing demand for tropical agricultural commodities has been responsible for the loss of massive areas of tropical forest and the emission of their carbon stocks into the atmosphere. In many places, this continues to be the case. However, sparked by concerns over global climate change and biodiversity loss, there is also a marked increase in demand for sustainably produced commodities that offers new opportunities for producers and countries willing to adopt new practices and put the necessary policies in place (Millard 2011). Prominent among those is a commitment to zero gross deforestation (Brown and Zarin 2013), which automatically confines new commodity expansion to already deforested lands (Dinerstein et al. 2014). Where these commodities are being produced in forest-like systems with native trees, this can amount to a re-agro-forestation strategy and lead to benefits not only for carbon storage, but potentially also for biodiversity.

We report here a case where through the coincidence of market and policy forces with favorable biophysical conditions, a commodity-driven re-agro-forestation frontier seems to be on the horizon in southeastern Pará in the Brazilian Amazon. A steadily increasing demand for cocoa on the global market that current producing regions may be unable to satisfy is coinciding with policies of the Brazilian government to enforce environmental laws requiring the restoration of excess deforested land with native trees. Since cocoa is a native tree of the Amazon forest, cocoa agroforests qualify for the task, and this has triggered increasing interest in cocoa planting among the region’s land owners. We show here that the potential of the region for producing cocoa may be globally significant, with already deforested areas with soils of naturally high fertility covering about 1.26 million hectares, enough to make a significant contribution to closing a looming gap in global cocoa supply of up to 1 million tons. The status of these soils after various periods under pasture use and the technologies and investments needed to establish tree crop agroforests on naturally high fertility soils of various states of degradation requires further study. Producing environmentally sustainable, deforestation free cocoa could become an additional and perhaps the principal basis for the livelihoods of thousands of land owners, especially family farmers, as well as their workers and share-croppers in this part of the “arc of deforestation” of the Brazilian Amazon. Where the rural population of southern Pará takes advantage of this opportunity, environmental benefits could also be substantial.

For the Brazilian cocoa sector that has been in a state of crisis since the decline of the Bahian cocoa production in the early 1990s under the joint influence of cocoa diseases and socioeconomic change (Alger and Caldas 1994), the emerging cocoa frontier in the Amazon presents an opportunity for re-entering the global cocoa market as a supplier of sustainable cocoa of a special kind: a commodity that has helped restore parts of the Amazon. This would no doubt be a novelty on the global cocoa market, and the global commodity market in general, and may attract followers in other parts of the tropics, and perhaps in other crops.^1^ For this to happen at a significant scale, policies need to be put in place at different levels in government, private sector and civil society to implement a strict zero-deforestation growth strategy for cocoa in the Amazon, which would effectively confine new cocoa planting to already deforested areas and make it a re-agro-forestation crop. At the same time, land owners willing to replant pasture or crop land in the Amazon with cocoa agroforests should receive support from government and market actors, including access to planting material, finance, training and technical advice, as well as marketing support. Finally, where cattle land is converted into other, more profitable and environmentally more desirable uses, this should be accompanied by the intensification of equivalent pasture areas to keep the output of animal products constant, lest cattle may be displaced to other areas and potentially cause indirect deforestation that would diminish or offset the environmental benefits of pasture re-agro-forestation.

## Footnotes

1 The Government of Ecuador has recently developed a policy to reforest several hundred thousand hectares of excess cleared pasture land with trees and tree crops, including cocoa.
